# Molecular Imaging of Induced Pluripotent Stem Cell Immunogenicity with *In Vivo* Development in Ischemic Myocardium

**DOI:** 10.1371/journal.pone.0066369

**Published:** 2013-06-20

**Authors:** Zhiqiang Liu, Xinyu Wen, Haibin Wang, Jin Zhou, Mengge Zhao, Qiuxia Lin, Yan Wang, Junjie Li, Dexue Li, Zhiyan Du, Anning Yao, Feng Cao, Changyong Wang

**Affiliations:** 1 Department of Advanced Interdisciplinary Studies, Institute of Basic Medical Sciences and Tissue Engineering Research Center, Academy of Military Medical Sciences, Beijing, P.R. China; 2 Department of Clinical Biochemistry, Chinese PLA General Hospital, Beijing, P.R. China; 3 Department of Cardiology, Xijing Hospital, Fourth Military Medical University, Xian, Shanxi, P.R. China; 4 Department of Chemical Engineering and Materials Science, Michigan State University, East Lansing, Michigan, United States of America; University of Tampere, Finland

## Abstract

Whether differentiation of induced pluripotent stem cells (iPSCs) in ischemic myocardium enhances their immunogenicity, thereby increasing their chance for rejection, is unclear. Here, we dynamically demonstrated the immunogenicity and rejection of iPSCs in ischemic myocardium using bioluminescent imaging (BLI). Murine iPSCs were transduced with a tri-fusion (TF) reporter gene consisting of firefly luciferase-red fluorescent protein-truncated thymidine kinase (fluc-mrfp-tTK). Ascorbic acid (Vc) were used to induce iPSCs to differentiate into cardiomyocytes (CM). iPSCs and iPS-CMs were intramyocardially injected into immunocompetent or immunosuppressed allogenic murine with myocardial infarction. BLI was performed to track transplanted cells. Immune cell infiltration was evaluated by immunohistochemistry. Syngeneic iPSCs were also injected and evaluated. The results demonstrated that undifferentiated iPSCs survived and proliferated in allogenic immunocompetent recipients early post-transplantation, accompanying with mild immune cell infiltration. With *in vivo* differentiation, a progressive immune cell infiltration could be detected. While transplantation of allogenic iPSC-CMs were observed an acute rejection from receipts. In immune-suppressed recipients, the proliferation of iPSCs could be maintained and intramyocardial teratomas were formed. Transplantation of syngeneic iPSCs and iPSC-CMs were also observed progressive immune cell infiltration. This study demonstrated that iPSC immunogenicity increases with *in vivo* differentiation, which will increase their chance for rejection in iPSC-based therapy.

## Introduction

Endogenous regenerative capacity of adult hearts is extremely limited, leading to increasing attention for exogenous regenerative strategies [1]. Cell transplantation strategies, often termed as “cellular cardiomyoplasty”, have provided ischemic heart diseases with novel therapies [2]. For the past few years, various cell types have been explored as seeding cells in cellular cardiomyoplasty. Among them, embryonic stem cells (ESCs) were considered as promising seeding cells due to their highly proliferative capacity and cardiomyogenic potential [3]–[5]. However, the therapeutic application of ESC was still hampered by immunological rejection and ethical conflicts [6].

Recently, a breakthrough was reported that ES-like cells were induced from mouse and human fibroblasts by forced expression of 4 transcription factors– Oct4, Sox2, c-Myc, and Klf4 [7], [8]. The derivation of induced pluripotent stem cells (iPSC) is not involved in the ethical conflict accompanying ESCs. Furthermore, derivation of patient-specific iPSC present a promising perspective for avoiding immunological rejection in cell-based therapy [9]. Lots of researchers have investigated the therapeutic potentials of iPSCs in various diseases, including sickle cell anemia, Parkinson’s disease and myocardial infarction *et al* [10]–[12]. Despite early encouraging results with iPSC transplantation, a latest report shows that iPS cell possessed certain immunogenicity and would be rejected by allogenic and syngeneic receipts [13], providing novel insights into the immunogenicity of iPSCs. However, the results from the study were mainly based on immunohistological evaluation, providing only a “snapshot” representation rather than time-dependent results of cell development/immune responses. Clearly, the limitations of immunohistological methods are unsuitable to dynamically present the immunogenicity of iPSCs during their *in vivo* development; the kinesis of iPSC immunogenicity with *in vivo* development has not yet been clarified.

For the past few years, several noninvasive imaging-based monitoring methods were developed, such as radionuclide imaging of cells labeled with F-18 fluoredeoxyglucose([18F]-FDG), magnetic resonance imaging (MRI) of cells labeled with iron oxide particles[14]–[16]. Though these imaging methods could provide information about graft location and quantity, their reliability and validity were restricted due to the shortfalls of these labeling agents, including decay of radioisotopes and signal dilutional effects during cellular division. [17]. Another imaging method reported is based on reporter gene, such as bioluminescence imaging (BLI) based on firefly luciferase (Fluc) gene and positron emission tomography (PET) imaging based on thymidine kinase gene(tTK)[17]–[20]. BLI is relatively convenient and low-cost, but restricted to small animals (low tissue penetration). PET imaging could be used in big animals and further translated into a clinical setting [17], [18].

In this study, murine iPSCs were efficiently transduced with lentivirus carrying a tri-fusion (TF) reporter gene consisting of Fluc, monomeric red fluorescent protein (mrfp) and tTK (fluc-mrfp-tTK ). To determine whether the differentiated state of iPSCs influences their immunogenicity, Ascorbic acid (Vc) was used to induce iPSCs into cardiac lineages. Then, undifferentiated iPSCs and iPS-derived cardiomyocytes (iPS-CM) were intramyocardially injected into allogenic murine model of MI. The *in vivo* fates of survival, proliferation and death of transplanted cells were assessed based on the TF reporter gene and noninvasive imaging platform. The immunogenicity of iPSCs with *in vivo* development was further evaluated by immunostaing based on immune cell infiltration. The immunogenicity of syngeneic iPSCs was also assessed.

## Results

### Efficient and Stable Transduction of Mouse iPSCs with TF Reporter Gene

**Figure 1:**
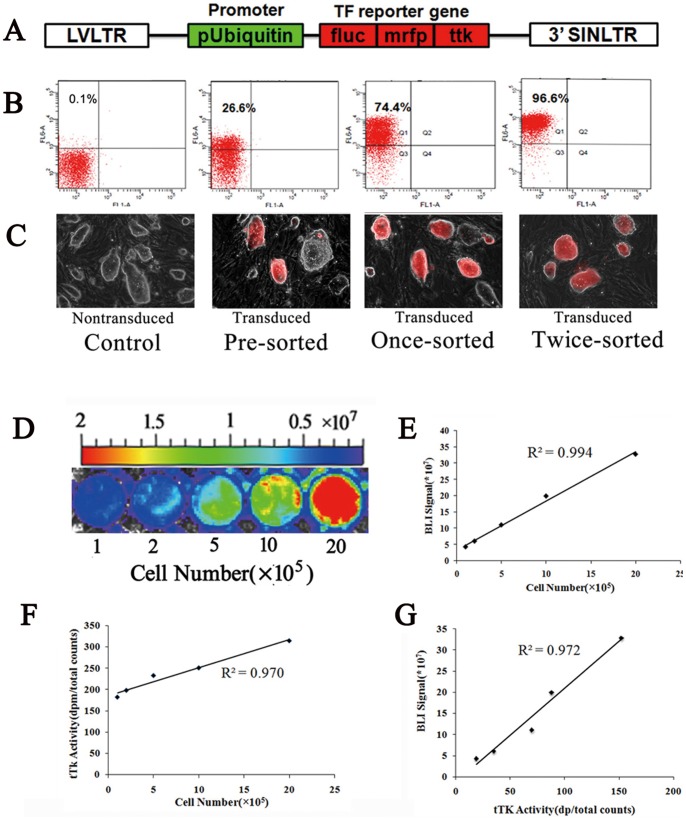
Lentiviral transduction of iPSCs. (A) lentiviral vectors consisting of tri-fusion reporter gene; (B) lentiviral transduction of iPS with tri-fusion reporter gene and sorting of successfully transduced cells; (C) RFP expression in transduced iPSCs; (D, E), iPS cells were seeded at a gradient density of 1, 2, 5, 10, 20×105/well for *in vitro* imaging. The linear relationship between iPSC-TF cells and bioluminescence signals (Fluc enzyme activity, R^2^ = 0.99); (F), The linear relationship between iPSC-TF cells and tTK enzyme activities (R^2^ = 0.97) was observed too. (G) Fluc and ttk activities also correlated well with each other (R^2^ = 0.97).

When using lentiviral vectors to transfect cells, several variables may influence transfection efficiency, including state of target cells, the activity of lentivirus and multiplicity of infection(MOI) used. In our study, after transduction with lentiviral vectors (Fig. 1A) at a MOI of 15, about 25–30% iPSCs(C57/129 line) were successfully transduced as analyzed by FACS. After twice sorting by FACS, the percentage of mrfp-positive cells was more than 95% (Fig. 1B), sorted iPSCs show bright mrfp expression under fluorescent microscope (Fig. 1C). *In vitro* analysis demonstrated nontransduced iPSC (control iPSC) and iPSC-TF showed similar morphology and expression of OCT4, SOX2, NANOG, SSEA-1 (Fig. S1A,B-a, B-b in File S1). FACS and MTT assay showed no significant difference between the 2 cell lines in cell viability and proliferation (Fig. S1B-c, B-d in File S1). Furthermore, the expression of TF reporter gene does not affect the tri-germ layer differentiation of iPSCs (Fig. S1B-e in File S1). The expression of TF reporter gene do not adversely affect the qualities and properties of iPSCs. During long term expansion, FACS analysis demonstrated relatively stable expression of reporter gene in iPSC-TF for up to 40 passages (Fig. S2A in File S1). BLI on the same number of iPSCs from different passages showed no significant difference either (Fig. S2B, C in File S1). *In vitro* bioluminescence imaging showed a strong linear relationship between cell number (from 10^5^ to 2×10^6^) and BLI signals (R^2^ = 0.99) (Fig. 1D, E). Similarly, a liner relationship between iPSCs and tTK activities was demonstrated (Fig. 1F, R ^2^ = 0.97). In addition, Fluc and tTK activities also correlated well with each other (Fig. 1G, R ^2^ = 0.97). These data suggest that both fluc and tTK reporters can be used to accurately quantify transplanted iPSCs and their progenies.

### Differentiation, Characterization of iPSC-derived Cardiomyocytes

The study shows that Vc could enhance cardiac differentiation of iPSCs (C57/129 line) in a dose-dependent manner (Fig. S3A in File S1), which was consistent with previous studies [21]–[23]. When the concentration of Vc was up to 10^−4^–10^−3^M, the percentage of contracting embryoid bodies (EBs) was significantly enhanced compared to that of control (*p*<0.01), indicating 10^−4^–10^−3^M Vc were optimal doses for cardiac differentiation of iPSC. During the differentiation, the number of contracting EBs increased with time and the maximum appeared at day 12–16 after differentiation. Apart from the increased number of contracting EBs, the contracting area was also expanded in induced differentiation of iPSC-EBs compared with that of control (*p*<0.01, Fig. S3B in File S1, control referred to spontaneous differentiation; induced differentiation referred to induced differentiation with 10^−3^ M Vc). Furthermore, the level of cardiac genes, α-MHC, GATA4, MLC-2v, were significantly higher in induced differentiation than that in spontaneous differentiation (*p*<0.01, Fig. S3C in File S1).

EBs containing contracting areas was immunostained with α-sarcomeric actinin and cardiac troponin T antibodies. Contracting areas were positively stained and the transverse striation could be observed under fluorescent microcopy (Fig. S3D in File S1). These results confirmed that Vc efficiently induced cardiac differentiation of iPSCs. iPSC-derivated non-contracting cells, contracting cells (Fig. S3E in File S1) and sorted iPSC-CMs (microdissected) (Fig. S3F in File S1) showed the stable expression of reporter gene. RT-PCR show that the expression of pluripotent genes decreased in differentiated iPSCs, while cardiac genes were significantly increased (Fig. S3 G, H in File S1). In microdissected iPSC-CMs, the pluripotent cells were much less and the ratio of cardiomyocytes were much higher (Fig. S3 G, H in File S1), counting the ratio of cTnT+ cells under fluorescent microscope showed that about 70–80% cells were cardiomyocytes.

### The Temporal Patterns of Allogenic iPSC Survival, Proliferation and Death in Ischemic Myocardium

**Figure 2:**
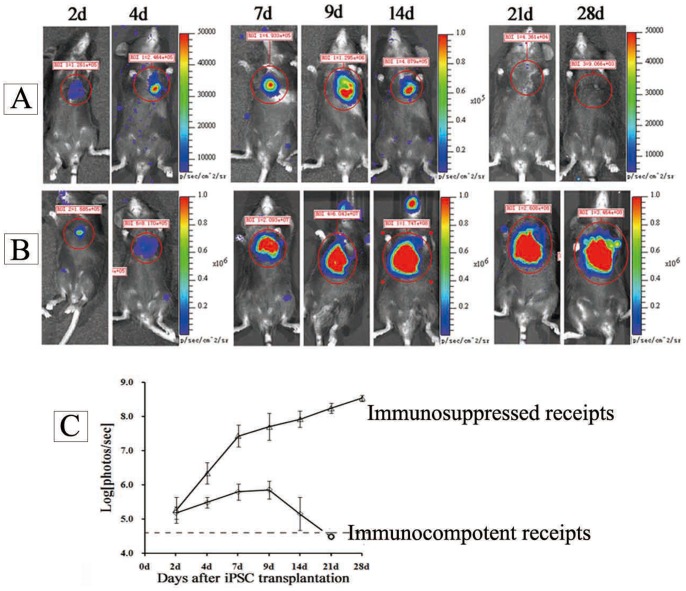
Noninvasive BLI of cardiac transplanted iPSCs. (A) imaging of intramyocardial transplanted iPSCs in immunocompetent allogenic recipients; (B) imaging of intramyocardial transplanted iPSCs in immunosuppressed allogenic recipients; (C) the *in vivo* growth curve of iPSCs in immunocompetent/immunosuppressed allogenic recipients (n = 6/group);

The BLI showed iPSCs survived in ischemic myocardium after transplantation. At 2 day after transplantation, visible BLI signals were detected in all mice receiving allogenic iPSC transplantation (1.62±0.63×10^5^ p/sec/mm^2^/sr). The signals increased with time until to a peak signal (about 7.7±6.4×10^5^ p/sec/mm^2^/sr) and then rapidly decreased to background at 2–3 weeks (Fig. 2A). These data indicated that transplanted iPSC survived and proliferated early after transplantation in ischemic myocardium (about before 10 days), then these cells underwent an acute lost (about 10 days later). Thus, BLI signals showed an evaluation at early stage and then a descent (Fig. 2C). In contract, in immune-suppressed mice with cyclosporin A (daily from one day before transplantation), the increases of BLI signals were maintained (Fig. 2B, C).

**Figure 3:**
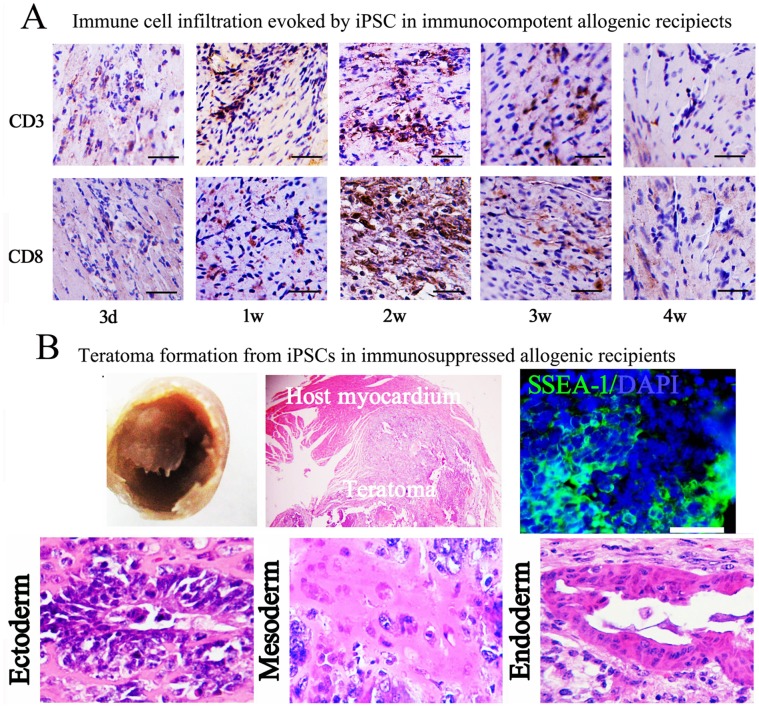
Immune cell infiltration and teratoma formation after allogeneic iPSC transplantation. **(**A) Allogeneic transplantation of iPSCs in immunocompetent allogeneic recipients evoked progressive infiltration of CD3, CD8+ immune cells (Bar = 25 µm); (B) Teratoma formation of iPSCs in immunosuppressed allogeneic recipientts.

To test the immune cell infiltrations, animals were sacrificed at day 3, 7, 14, 21 and 28 after receiving iPSC transplantation (n = 6/group). In sections from day 3 samples, mild immune cell infiltrations were observed (CD3: +; CD8: +/−; Fig. 3A, table 1). In sections from day 7 samples, most samples were detected a moderate infiltration of inflammatory cells, significantly more CD3, CD8+ lymphocytes were observed in immunostained sections (CD3:++; CD8:++, Fig. 3A, table 1) compared with day 3 samples. In sections from week 2 samples, massive infiltration of CD3 and CD8+ T cells was observed (CD3: +++; CD8+++; Fig. 3A, table 1), indicating vigorous immune rejection of recipients(sham animals that received PBS injection were used to exclude unspecific immune cells due to tissue damage, Table S2 in File S1). At day 21 and day 28, infiltrating immune cells decreased again (Fig. 3A, table 1), as most of exogenous cells have been rejected. These data were in accordance with the BLI data, which increased at early time (about within 7–10days) and rapidly decreased after that. In recipients with immunosuppression, visible teratomas were formed in myocardium (Fig. 3B). Immunostaining showed that pluripotent cells expressing SSEA-1 existed inside teratomas, indicating that teratomas were keeping growing (Fig. 3B). The result was also in accordance with the increasing signals observed in BLI. From H&E stained sections, typical structures of tri-germ layers could be observed (Fig. 3B).

**Table 1:** Infiltrating inflammatory cells with time after iPSC transplantation into ischemic hearts of immunopotent mice(n = 4 for each detection time).

Inflammatory Cells	Time after cell transplantation				
	3d	7d	14d	21d	28d
**CD3**	+	++	+++	++; +	–
**CD8**	+/−	++	+++	++; +	–

CD3: Cell surface markers of T lymphocytes, CD8: cytotoxic T cells;. Degree of infiltration:- absent; +/−, trace; +, mild; ++, moderate; and +++, severe.

### Immune Cell Infiltration After Syngeneic iPSCs Transplantation in Ischemic Myocardium

**Figure 4:**
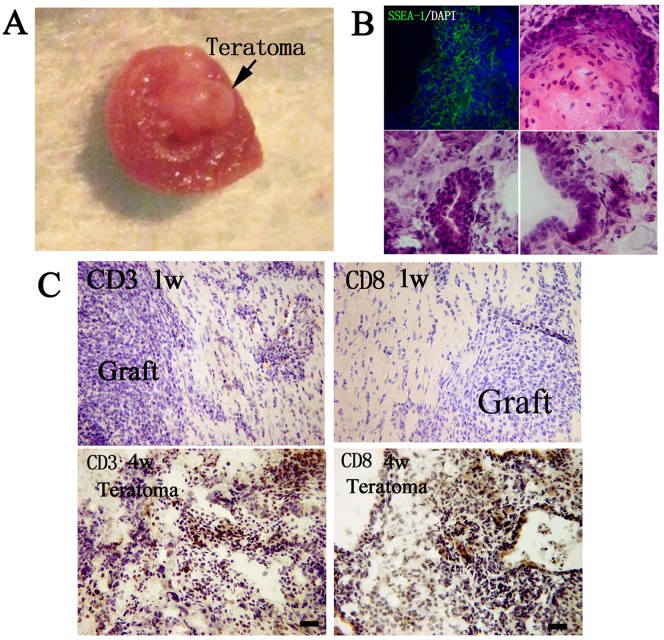
Teratoma formation and immune cell infiltration after syngeneic iPSC transplantation. **(**A), iPSCs in ischemic syngeneic hearts formed visible teratomas 4w after transplantation; (B), Pluripotent cells expressing SSEA-1 and typical tri-germ layer structures could be observed in teratomas; (C), immune cell infiltration inside the graft, 1w: immune cell infiltration was observed; 4w: significant immune cell infiltration was detected, indicating syngeneic iPSCs also evoked recipient immune rejection with *in vivo* development. (Bar = 50 µm).

The immunogenicity of syngeneic iPSCs (C57 cell line) in mouse ischemic myocardium (C57 mice) was also detected to provide additional information. To ensure as far as possible the syngeneicity of transplanted cells, syngeneic iPSCs were not genetically modified with reporter gene. After cell transplantation, histology and immunostaing were used to detect the survival of grafts and immune rejection. 1w after iPSCs transplantation in syngeneic receipts, we did not detect apparent immune cell infiltration (data not shown). At 4w, visible teratomas were formed like that in immunosuppressed allogenic recipients. Pluripotent cells and typical structures of tri-germ layers were also detected inside syngeneic teratomas. After immunostaining against lymphocyte, we found apparent immune cell infiltration occurred inside syngeneic teratomas (Fig. 4). The results provided additional evidence for increasing immunogenicity of iPSCs with development in ischemic myocardium.

### The Temporal Patterns of Allogenic iPSC-CMs Survival, Proliferation and Death in Ischemic Myocardium

**Figure 5:**
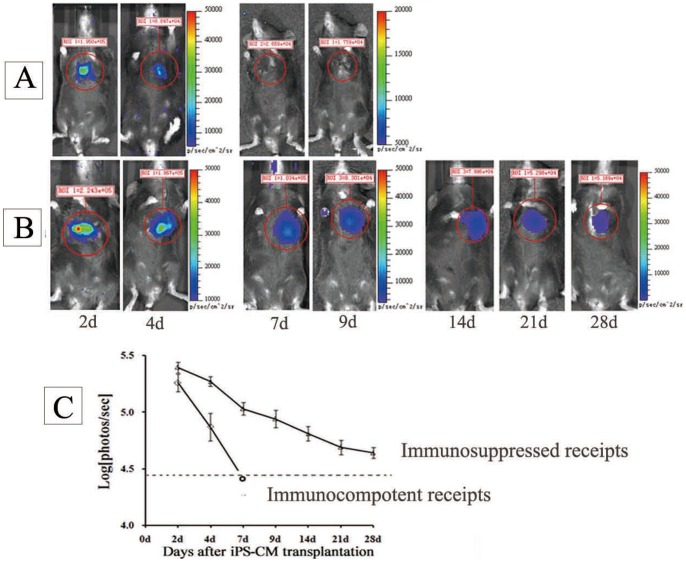
Noninvasive BLI of cardiac transplanted iPSC-CMs. (A) Imaging of intramyocardial transplanted iPSC-CMs in immunocompetent allogenic recipients; (B) imaging of intramyocardial transplanted iPSC-CMs in immunosuppressed allogenic receipts; (C) the *in vivo* BLI signals of iPSC-CMs in immunocompetent/immunosuppressed allogenic recipients(n = 6/group).

To further test whether *in vivo* development or differentiation increase iPSC immunogenicity, reporter-expressing iPSC-CMs, which were derived from C57/129 iPS-TF cells, were intramycardialy injected into immunocompetent allogenic C57 mice of MI and compared with undifferentiated C57/129 iPSCs. A sharp decrease in BLI signals was observed (Fig. 5A, C) after iPSC-CM transplantation. As shown in Fig. 5, almost all the transplanted cells were rejected within 2–4days, resulting the sharp decrease in BLI signals from the beginning of the detection. When the mice were immunosuppressed, the decrease of BLI signals with time was significantly attenuated (Fig. 5B, C). The results suggested a higher immunogenicity of iPSC-CMs and an acute immune-rejection evoked by them.

**Figure 6:**
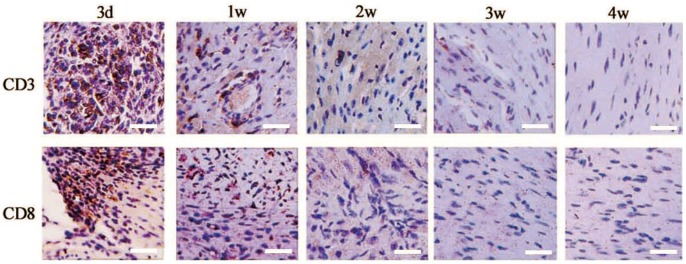
Immune cell infiltration after allogeneic iPSC-CM transplantation. Allogeneic transplantation of iPSC-CMs in immunocompetent recipients evoked acute infiltration of CD3, CD8+ immune cells. (Bar = 25 µm).

Evaluation of immune cell infiltrations after iPSC-CM transplantation provided further evidence that differentiated iPSCs possess a higher immunogenicity. Different from undifferentiated iPSC, transplantation of iPSC-CMs elicited an acute immune rejection, massive infiltrating immune cells (CD3 and CD8+ cells) were observed at the first detection (at 3day, Fig. 6, Table 2), suggesting a higher immunogenicity of iPS-CMs. This is in accordance with the sharp loses of iPSC-CM grafts.

**Table 2:** Infiltrating inflammatory cells in myocardium after Injection of Cardiomyocytes induced from iPSCs in immunopotent mice(n = 4 for each detection time).

Inflammatory Cells	Time after cell transplantation				
	3d	7d	14d	21d	28d
**CD3**	+++	+; ++	+/−	+/−	–
**CD8**	+++	+; ++	+/−	–	–

CD3: Cell surface markers of T lymphocytes, CD8: cytotoxic T cells;. Degree of infiltration:- absent; +/−, trace; +, mild; ++, moderate; and +++, severe.

Taken together, these findings suggest that undifferentiated iPSCs possess a low immunogenicity and may be of immune privilege in ischemic myocardium of allogenic receipts, but once they reach a more differentiated state, their immunogenicity increase and can be effectively recognized and immediately rejected by the recipient immune system. Therefore, transplantation of undifferentiated iPSC in ischemic myocardium will elicit a progressive immune rejection with their *in vivo* development, as the process will result in a more differentiated status and a higher immunogenicity of differentiated cells.

### Immune Cell Infiltration After Syngeneic iPSC-CM Transplantation in Ischemic Myocardium

**Figure 7:**
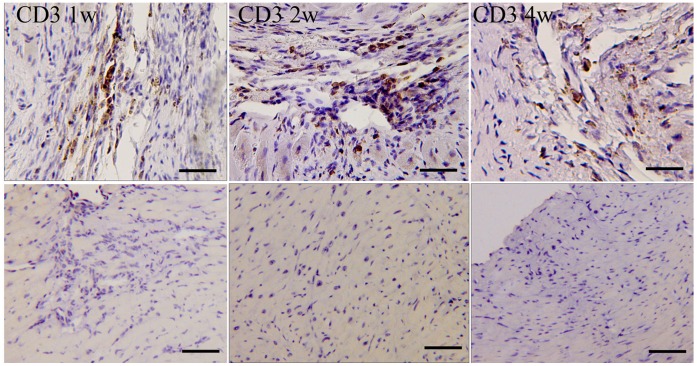
Immune cell infiltration after syngeneic iPSC-CM transplantation. Upper ones: Mice with syngeneic iPSC-CM transplantation; Below ones: Control without cell transplantation. (Bar = 50 µm).

After syngeneic iPSC-CM transplantation, immunostaing against CD3 were performed at 1w, 2w and 4w to detect the immune rejection. 1w after iPSC-CM transplantation in syngeneic receipts, apparent immune cell infiltration was observed. At 2w, the immune cell infiltration was even more severe. The results demonstrated that iPSC-CMs evoked more sever immune rejection in syngeneic receipts than undifferentiated iPSCs(Fig. 7). At 4w, the immune cell infiltration was attenuated. This may be because that most of iPSC-CMs have been lost.

## Discussion

Using non-invasive imaging technologies, this study was designed to investigate the immunogenicity of iPSC with *in vivo* differentiation in ischemic myocardium. The data showed that allogenic transplanted iPSC in ischemic myocardium displayed a growth curve with an early elevation and a subsequently acute descent, indicating a process of survival, proliferation and death of iPSC with *in vivo* development. Immunostainning demonstrated there is progressive infiltration of immune cells within the iPSC graft after intramyocardial transplantation. The most vigorous infiltration of immune cells was observed during the acute descent of BLI signals, followed by disappearance (BLI signals) of the iPSC allografts. The finding suggests that undifferentiated iPSCs possess a low immunogenicity and may escape the immune system of allogenic recipients. However, the immunogenicity will increase with iPSC development in ischemic myocardium, resulting in an immediate recognition by recipient immune system and an acute immune rejection.

Transduction with TF-reporter gene has been well established in ESC [17]. The labeling with tri-fusion reporter gene could meet the tracking demands for various purposes and in different settings. Precisely, the mrfp could be used as a marker for sorting successfully transduced iPSCs by FACS, as well as for histological detection by fluorescent microscopy; The fluc could be used for BLI of iPSC fate longitudinally, but it is not suitable for big animals due to the low tissue penetration of its signals. PET imaging based on tTK reporter is not constrained by tissue depth and therefore suitable for both small and big animals and has the potential to translate into future human trials with clinical PET [17]. However, PET imagings are complex to carry out and require radiotracer. More work will be needed in the image processing and data analysis. In this study, mice were used as models, it is why only BLI was performed along most time points.

Based on bioluminescence imaging, we demonstrated the BLI signals of transplanted iPSCs, indicting the kinetics of iPSC survival, proliferation and death in allogenic ischemic myocardium. At the first time points (2 days), the BLI signals were comparable between animals with and without immune suppression, the infiltration of immune cells (CD3 and CD8+ cells) were few (3 days), suggesting that undifferentiated iPSCs are of a low immunogenicity in allogenic recipients. Though BLI signals showed an elevation in the following time, progressive infiltration of immune cells were observed, indicting an increasing immune responses. After a peak in BLI signals, rapid decrease appeared and vigorous infiltration of inflammatory cells were observed.

One could argue that death of iPSC could be due to hostile environment caused by ischemia (e.g., hypoxia, denutrition) rather than receipt immune rejection, but immune suppression maintained the BLI signal increase in the whole experiment, suggesting that immune rejection was the main reason of iPSC demkse. One could also argue that death of iPSC may be due to inflammation caused by ischemia-associated injury. However, no evident death of iPSC was observed early after transplantation, while an immediate death of iPSC-CMs was observed upon transplantation. Therefore, a reasonable explanation for BLI curve and progressive infiltration of immune cells was that undifferentiated iPSC may possess a low immunogenicity which may escape the immune recognization of allogeneic recipients, but the immunogenicity of iPSC increases with their *in vivo* development and differentiation, that is, differentiated iPSC possess a higher immunogenicity, evoking acute rejection of recipits.

It has been demonstrated that iPSC would also elicite immune response in syngeneic recipients by previous study [13], but teratoma formation could still be observed even 2 months after transplantation, suggesting immune recognition of iPSC immunogenicity by syngeneic recipients was relatively insensitive. In this study, though syngeneic could proliferate in receipt ischemic myocardium and even formed teratomas, significant immune cell infiltrations have been detected inside teratomas at 4w, indicating that syngenic iPSC immunogenicity was also increased with *in vivo* development in ischemic myocardium. In BLI study, allogenic receipts were used so that increased immunogenicity of iPSC could be sensitively and immediately recognized by recipientpt immune system.

To circumvent immune recognition by the receipt, three strategies were usually adopted, including syngeneic transplantation, immunosuppressive therapy and transplantation into an immune privileged site[24]–[26]. Clearly, syngeneic transplantation are unfeasible to circumvent iPSC-elicited immune responses [13]. Therefore, transplantation of allogeneic or autologous iPS/iPS-derivatives for treatment of MI might require immunosuppressive therapy. However, we demonstrated that immune suppression would result in a high risk of teratoma formation from iPSCs. Furthermore, the teratoma may metastasize in recipients like tumors, this is a common concern faced by ESC and iPS [6], [27], [28]. Therefore, in the application of iPSCs or ESCs,suitable strategies to reduce the tumoragenic risk in addition to immune suppression would be required, e.g.,pretreatment of cells with metformin or etoposide [29], [30]. Transplantation of iPSC-CMs may be a possible way to avoid teratoma formation [28]. Though several methods have been reported to differentiate iPSC into CMs[31]–[33], few have been used to produce iPS-CMs for cardiac transplantation, possibly due to high expense, cytotoxicity of inducers, complicated protocols, *et al*. In the study, we developed an efficient method for producing iPS-CMs. The inducer in the study, Vc, is cheap and free of cytotoxicity, thus could be used in large scale at a low cost. Why Vc induce cardiac differentiation from iPSCs remains to be evaluated in detail.

In conclusion, based on the imaging platform, we firstly demonstrated the progressing immunogenicity of iPSCs with their development in allogenic ischemic myocardiunm, that is, undifferentiated iPSCs may be of immune privilege in allogeneic recipients and their immunogenicity will increase upon differentiation. In addition, we established an efficient method for producing iPS-CMs.

## Materials and Methods

### Generation and Culture of Undifferentiated Murine iPSCs

129/C57 iPSC line iPS-tet-B3 (Allogeneic) was a kind gift of Dr Gang Pei and Jiuhong Kang [34]. C57 iPSC line (syngeneic) was purchased from Shang Hai SIDANSAI Biotechnology CO., LTD (www.sidansai.com). Undifferentiated iPSCs were cultured on the top of mouse embryonic fibroblasts feeder layer inactived by 10 ug/mL mitomycin C (Sigma). The cells were cultured on 0. 1% gelatin (Sigma)-coated plastic dishes in Dulbecco’s modified Eagle medium(Gibco) supplemented with 15% FBS(Gibco), 0.1 M beta-mercaptoethanol(Gibco), 1×glutamine(Gibco, 25030), 1×MEM non-essential amino acid(Gibco, 25030), and 1000 U/mL leukemia inhibitory factors(Chemicon). The medium was changed every day. Undifferentiated iPSCs were passaged onto new feeder layers every 2–3 days at the ration of 1∶3–1∶6.

### Production of Lentiviral Vectors Carrying Tri-fusion Reporter Gene and Establishment of iPS-TF Line

Three plasmids used for the production of TF reporter gene-carrying lentivius (LV-fluc-mrfp-ttk), plasmid containing Flu-mrfp-tTK fusion reporter gene, packaging system ps PAX2 and envelop plasmid pMD2G, were kindly provided by Sanjiv Sam Gambhir (from Stanford University, Radiology Department) [17]. Production of lentiviral vectors carrying tri-fusion reporter gene and transduction of iPSCs see Supplementary Materials in File S1.

### *In vitro* Characterization of TF Reporter Gene-expressing iPSCs

To determine the influence of reporter genes on iPSCs, the viability, proliferation and pluripotency of iPSC-TF were analyzed and compared with control iPSC (untransduced iPSCs). Details see Supplementary Methods in File S1.

### *In vitro* Optical Bioluminescence Imaging

For *in vitro* bioluminescence imaging, 100 uL iPSC-TF suspensions containing different cells were added to wells of a 48-well or 96-well plate. A 200× luciferin stock solution (30 mg/ml, Sciencelight) in sterile water was prepared and freezed in −20°C. Before imaging, the 200×stock solution was diluted to 2×solution using culture medium. To every 100 uL culture medium 100 uL 2×solution D-Luciferin reagent were added at room temperature. The bioluminescent signals generated by iPSC-TF were detected by a highly sensitive charge-coupled device (CCD) camera (IVIS 50, Xenogen, USA).

### *In vitro* Assay of tTK Activity

For assay of tTK activity, FHBG accumulation assays were performed on various numbers of iPS-TF according to the previously report [35]. Cells were seeded in 48-well plate, thirty-seven kilo becquerels of FHBG was added to each well and the cells were incubated with the tracer for 1 hour at 37°C. After removing the tracer, cells were washed three times with PBS. Then, the cells were lysed with 150 µL of 1N NaOH. Cell lysates were analyzed in a gamma counter.

### Derivation of iPSC-cardiomyocytes

To initiate embryonid body formation, iPS cells were suspended in differentiation medium without LIF and transferred into low adherent petri dishes. 10 mL suspensions containing 1×10^6^ iPSCs were transferred into each petri dishes (100 mm diameter). Medium was changed every two days, iPSCs aggregate and grow in suspension to form EBs.

5 days’ EBs were transferred onto 0.1% gelatin-coated tissue culture dishes, ascorbic acid (Vc) was used as inducer to induce cardiac differentiation from mouse iPSCs. To optimize inducing efficiency, 0, 10^−7^, 10^−6^, 10^−5^, 10^−4^, 10^−3^ M Vc were used and compared. The Vc was added when 5-day EBs were transferred onto 0.1% gelatin-coated culture dishes and in the whole process of differentiation.

### MI Model and Cell Transplantation

Female C57BL/6 mice (8–10 weeks) were purchased from the Experimental Animal Center, Academy of Military Medical Science (Beijing, PRC). The Institutional Animal Care and Use Committee (IACUC) of the Chinese Academy of Military Medical Science, Beijing, China, approved all experiments in this study. Every effort was made to minimize animal suffering and the number of animals used. Animals were maintained at a temperature of 24±2°C with a normal day–night cycle with free access to water and food.

Myocardial infarction models were produced in 8–10 week old female C57BL/6 mice as previous reports [12]. Briefly, mice were anesthetized with sodium pentobarbital (100 ug/g body weight) and intubated. After left thoracotomy, the pericardial membrane was removed and the proximal left anterior descending coronary artery (LAD) was ligated to induce MI. iPSCs were trypsinized and prepared as single cell suspension in PBS. For allogenic C56/129 iPSC line, 10 uL cell suspensions (500000 iPSCs or 1000000 iPSC-CMs) were injected into the border of infarct area at a single site (n = 26/group). The mice receiving allogenic iPSC or iPSC-CM injection (C57/129 iPSC line) were further divided two group: immunopotent group and immuno-suppressed group with cyclosporin A (20 mg/kg/d i.p. Ciclosporin A, daily from one day before transplantation). For syngenic C57 iPSC line, 10 uL cell suspensions (500000 iPSCs, n = 8, or 1000000 iPSC-CMs, n = 12) were injected into the border of infarct area at a single site. Sham animals had induced MI and were injected with PBS (n = 20). Successful injection was typified by the formation of a bleb covering the inject zone. Then the chest wall was closed and the mice were allowed to recover under care.

### Bioluminescence Imaging of iPSC Transplant

Noninvasive imaging was longitudinally performed for 4 weeks. BLI was performed at day 2, 4, 7, 9, 14, 21, 28 time points post surgery. After intraperitoneal injection of the reporter probe D-Luciferin (150 mg/kg), animals were anesthetized with inhaled isoflurane (2% to 3%) and imaged for 1 to 10 min until the maximum signals were obtained. Bioluminescence signals were quantified in units of maximum photons per second per centimeter square per steridian (p/s/cm^2^/sr) as previously described [17], [36].

### Histology and Immunohistochemical Staining

For immunohistochemical staining, embryonic stem cell marker, OCT4, SOX2, NANOG and SSEA-1 were detected to determine the undifferentiated state of iPSCs. Cardiac markers, α-Sarcomeric actinin and cTnT were detected to determine differentiated cardiomyocytes. To evaluate immune cell infiltration, mice receiving iPSC/iPS-CM were sacrificed; hearts were explanted and fixed in 4% paraformaldehyde. 5 µm paraffin-embed sections were prepared and immunostaining was performed with anti-CD3 and CD8 antibodies (abcam) (CD3: cell surface markers of T lymphocytes; CD8: cytotoxic T cells). Details see Supplementary Methods in File S1. The infiltration was divided into 4 degrees according to the previous report [37]: - indicates no infiltration; +/−, few infiltration; +, scattered infiltration; ++, modest infiltration; and +++, vigorous infiltration. After immunohistochemical staining, sections were evaluated and graded a score for degree of immune cell infiltration.

### RNA Extraction and RT-PCR

Total RNA was extracted with RNAprep pure Cell/Bacteria Kit (TIANGEN) according to manufacturer’s instruction. Reverse transcription were performed using standard procedures to synthesize first-strand cDNA. In the following PCR amplification, each cycle consisted of denaturation at 94°C for 30 seconds, annealing at 55°C for 30 seconds and extension at 72°C for 45 seconds.

Polymerase chain reaction was performed for 35 cycles. To quantify the gene expression, the signal intensity of the amplification product was normalized to its respective GAPDH signal intensity. Primers used in PCR amplification were listed in the Table S1 in File S1.

### Statistical Analysis

All experiments were performed at least 3 times, and data were expressed as mean±standard deviation and analyzed by Student *t* test using SPSS17.0. A value of *P*<0.05 was considered statistically significant.

## Supporting Information

File S1 **Table S1.** Primers used in the PCR. **Table S2.** Infiltrating inflammatory cells in MI mice receiving PBS injection (n = 4 for each detection time). **Figure S1. Characterization and Comparison between control iPS and iPS-TF cells.** Both iPS and iPS-TF colonies cells were positive for pluripotent ESC markers – SOX2, SSEA-1, NANOG and OCT4. In iPS-TF colonies, colocalization of mrfp and ESC markers were observed under a fluorescent microscope, while in iPS colonies, no expression of mrfp was observed. The nucleus were stained with hochest (Bar = 100 µm) (A). The expression of ESC markers were also analyzed in gene level by RT-PCR, no significant difference were observed between the control iPS and iPS-TF cells(B-a, B-b); Cell viabilities and proliferation of iPS and iPS-TF cells were analyzed and compared within 72 h culture. Quantification of viable cells at 24 h, 48 h and 72 h time points showed no significant difference between iPS and iPS-TF cells(B-c); No significant difference in proliferation capacities was observed between iPS and iPS-TF cells either(B-d); After EB formation and differentiation, both iPS and iPS-TF cells could differentiate into tridermic lineages(B-e). **Figure S2. Stable expression of repoter gene in iPSC-TF cells.** (A), Stable expression of repoter gene during long-term passage of iPSCs; (B, C), Bioluminescence assay on the same number (10^5^) of iPSC-TF cells from different passages. **Figure S3. Differentiation and characterization of iPSC-CMs.** (A), Comparison of the contracting areas (Bar = 100 µm). (C) The expression of cardiac genes in Vc-induced/spontaneous differentiation of iPSCs; (D) Contracting EBs were positively stained by cardiac-specfic antibodies. (Bar = 50 µm). (E) Expression of reporters in iPSC-noncontracting and contracting derivates; (F), Expression of reporters in iPSC-CMs(Bar = 50 µm); (G) The expression of pluripotent, cardiac genes in iPSCs, iPSC-cardiac derivates and iPSC-CMs. Lane 1: iPSCs; Lane 2: iPSC-cardiac derivates; Lane 3: iPSC-CMs; (H) Comparison of iPSCs, iPSC-cardiac derivates and iPSC-CMs. *p<0.01. **Supplementary Methods.** Production of lentiviral vectors carrying tri-fusion reporter gene and establishment of iPS-TF line.
(DOC)
