# Supplementary material for: Molecular Imaging of Induced Pluripotent Stem Cell Immunogenicity with In Vivo Development in Ischemic Myocardium
Source: PLoS One. 2013 Jun 20;8(6):e66369. doi: 10.1371/journal.pone.0066369 (PMC3688792; doi:10.1371/journal.pone.0066369)
Supplement: File S1 — Table S1. Primers used in the PCR. Table S2. Infiltrating inflammatory cells in MI mice receiving PBS injection (n = 4 for each detection time). Figure S1. Characterization and Comparison between control iPS and iPS-TF cells. Both iPS and iPS-TF colonies cells were positive for pluripotent ESC markers – SOX2, SSEA-1, NANOG and OCT4. In iPS-TF colonies, colocalization of mrfp and ESC markers were observed under a fluorescent microscope, while in iPS colonies, no expression of mrfp was observed. The nucleus were stained with hochest (Bar = 100 µm) (A). The expression of ESC markers were also analyzed in gene level by RT-PCR, no significant difference were observed between the control iPS and iPS-TF cells(B-a, B-b); Cell viabilities and proliferation of iPS and iPS-TF cells were analyzed and compared within 72 h culture. Quantification of viable cells at 24 h, 48 h and 72 h time points showed no significant difference between iPS and iPS-TF cells(B-c); No significant difference in proliferation capacities was observed between iPS and iPS-TF cells either(B-d); After EB formation and differentiation, both iPS and iPS-TF cells could differentiate into tridermic lineages(B-e). Figure S2. Stable expression of repoter gene in iPSC-TF cells. (A), Stable expression of repoter gene during long-term passage of iPSCs; (B, C), Bioluminescence assay on the same number (105) of iPSC-TF cells from different passages. Figure S3. Differentiation and characterization of iPSC-CMs. (A), Comparison of the contracting areas (Bar = 100 µm). (C) The expression of cardiac genes in Vc-induced/spontaneous differentiation of iPSCs; (D) Contracting EBs were positively stained by cardiac-specfic antibodies. (Bar = 50 µm). (E) Expression of reporters in iPSC-noncontracting and contracting derivates; (F), Expression of reporters in iPSC-CMs(Bar = 50 µm); (G) The expression of pluripotent, cardiac genes in iPSCs, iPSC-cardiac derivates and iPSC-CMs. Lane 1: iPSCs; Lane 2: iPSC-cardiac derivates; Lane [file pone.0066369.s001.doc]

**Supplementary materials**

**Manuscript Title:** Molecular Imaging of iPSC Immunogenicity With *In Vivo* Development in Ischemic Myocardium

**Supplementary methods**

**Production of lentiviral vectors carrying tri-fusion reporter gene and establishment of iPS-TF line**

Three plasmids used for the production of TF reporter gene-carrying lentivius (LV-fluc-mrfp-ttk), plasmid containing Flu-mrfp-tTK fusion reporter gene, packaging system ps PAX2 and envelop plasmid pMD2G, were kindly provided by Sanjiv Sam Gambhir (from Stanford University , Radiology Department). For lentivirus production , these plamids were cotransfected into 293T cells using Entranster^TM^–H reagent (Engreen Biosystem Co, Ltd.) according to the manufacturer’s instruction. Lentivirus supernatant was harvested 72 hours after transfection and concentrated in ultrafiltrate centrifuge tubes at 5000 g. Concentrated virus was titrated on 293T cells and freezed in -70°C for future use.

Mouse iPS-tet-B3 cells were transduced with LV-pUb-fluc-mrfp-tTK at a multiplicity of infection (MOI) of 15. Briefly, 5×10^5^ iPS cells (per well) were seeded on 6-well plate 12h before transfection. Before transduction, the culture medium was replaced by 1mL OptiMEM, then lentiviras was added and incubated overnight in 37℃,5% CO_2_. After that, the lentiviras was removed and fresh medium (H-DMEM supplemented 20% serum) was added. The infectivity was determined by mrfp expression as analyzed on FACScan (BD FACSVantage Diva). Successfully transduced cells were sorted by fluorescence-activated cell sorting (FACS) based on the expression of mrfp. The 10% highest mrfp expressing cells were sorted and reseeded on feeder layers. If once sorting is not enough, a second or third sorting would be needed until purified iPS-TF cell line was established.

***In vitro* characterization of TF reporter gene-expressing iPSCs**

To determine the influence of reporter genes on iPSCs, the viability, proliferation and pluripotency of iPSC-TF were analyzed and compared with control iPS (untransduced iPSCs).

For cell viability analysis, iPSC-TF and control iPSCs were seeded on 0.1% gelatin-coated 6-well culture plates. At time points of 24h, 48h, and 72h after seeding, cells were harvested and prepared as single cell suspensions. After being stained with propidiumiodide (PI), cell viabilities were analyzed by flow cytometry.

For proliferation analysis, iPSC-TF and control iPSCs were seeded on 0.1% gelatin-coated 96-well culture plates. At time points of 24h, 48h, and 72h after seeding, cells were stained by MTT, optical density values were measured using a spectrophotometer at 492 nm wavelength. For pluripotency analysis, the expression of ESC markers in iPS-TF, including OCT4, SOX2, NANOG, SSEA-1, were determined by immunostaining with corresponding antibodies and RT-PCR using corresponding primers. The details for immunostaining are described below.

**Histology and immunohistochemical staining**

For immunohistochemical staining, embryonic stem cell marker, OCT4, SOX2, NANOG and SSEA-1 were detected to determine the undifferentiated state of iPSCs. Cardiac markers, α-Sarcomeric actinin and cTnT were detected to determine differentiated cardiomyocytes.

Briefly, iPSC clones and differentiated iPS-EBs grown in slides were washed and fixed with 4% paraformaldehyde in 0.1M phosphate buffer (pH 7.4). After permeabilization with 0.1% Triton X-100, the samples were incubated with the primary antibodies against OCT4, SOX2, NANOG and SSEA-1, or cTnT ( Sigma) and α-sarcomeric actinin (Sigma) overnight at 4◦C. FITC-labeled goat anti-mouse IgG (or IgM) was used as the secondary antibody. The cells were incubated with Hoechst33258 for genomic DNA staining and observed under a fluorescent microscope (Olympus Optical, Melville, NY).

To evaluate immune cell infiltration, mice receiving iPSC/iPS-CM were sacrificed; hearts were explanted and fixed in 4% paraformaldehyde. 5μm paraffin-embed sections were prepared and immunostaining was performed with anti-CD3 and CD8 antibodies (abcam). Sections were blocked and incubated with primary antibodies overnight at 4 °C. After immunohistochemical staining, sections were evaluated and graded a score for degree of immune cell infiltration according to the previous report. The infiltration was divided into 4 degrees: - indicates no infiltration; +/-, few infiltration; +, scattered infiltration; ++, modest infiltration; and +++, vigorous infiltration.

**Supplemeetary Table S1:** Primers used in the PCR

| **Genes** | **Primers** |
| --- | --- |
| **activinβ** | 5’-GTCAATTTGACGTGGTTTCC-3’  5’-GCAAGAATGTGCTGATCAAC-3’ |
| **MLC-2v** | 5’-GCC AAG AAG CGG ATA GAA GG-3’  5’-CTG TGG TTC AGG GCT CAG TC-3’ |
| **Nkx2.5** | 5’-AGC AAC TTC GTG AAC TTT G--3’  5’-CCG GTC CTA GTG TGG A-3’ |
| **a-MHC** | 5’- ACC GTG GAC TAC AAC AT-3’  5’- CTT TCG CTC GTT GGG A-3’ |
| **β-MHC** | 5’- ACC CCT ACG ATT ATG CG-3’  5’-GTG ACG TAC TCG TTG CC-3’ |
| **ANF** | 5’-GGGGGTAGGATTGACAGGAT-3’  5’-CAGAGTGGGAGAGGCAAGAC-3’ |
| **Oct4** | 5’- GGAGGAAGCCGACAACAATGAG-3’  5’-TGGGGGCAGAGGAAAGGATACAG-3’ |
| **Sox2** | 5’-CCAAGACGCTCATGAAGAACG--3’  5’-GGAGTGGGAGGAAGAGGTAAC-3’ |
| **Nanog** | 5’- CTGGTCCCCACAGTTTGCCTA-3’  5’- CTGGTCCCCACAGTTTGCCTA-3’ |
| **GATA4** | 5’- GAAAACGGAAGCCCAAGAACC-3’  5’-TGCTGTGCCCATAGTGAGATGAC-3’ |
| **TTR** | 5’CTCACCACAGATGAGAAG-3’  5’-GGCTGAGTCTCTCAATTC-3’ |
| **AFP** | 5’-TCGTATTCCAACAGGAGG-3’  5’-AGGCTTTTGCTTCACCAG-3’ |
| **Neurofilament** | 5’-TGGCTTAGATGTGAGCCCTG -3’  5’-CTATGGCGTGTGAAGTGACC -3’ |
| **collagen V** | 5’-CAAGCATAGTGGTCCGAGTC-3’  5’-AGGCAGGTCAAGTTCTAGCG-3’ |
| **GAPDH** | 5’AAC GAC CCC TTC ATT GAC-3’  5’-TCC ACG ACA TAC TCA GCAC-3’ |

**Supplementary Table S2:** Infiltrating inflammatory cells in MI mice receiving PBS injection (n=4 for each detection time)

| **Inflammatory Cells** | **Time after cell transplantation** | | | | |
| --- | --- | --- | --- | --- | --- |
|  | **3d** | **7d** | **14d** | **21d** | **28d** |
| **CD3** | +/- | +/- | - | - | - |
| **CD8** | +/- | - | - | - | - |

CD3: Cell surface markers of T lymphocytes, CD8: cytotoxic T cells; . Degree of infiltration:- absent; +/-, trace; +, mild; ++, moderate; and +++, severe.

**Supplementary Figures**

**
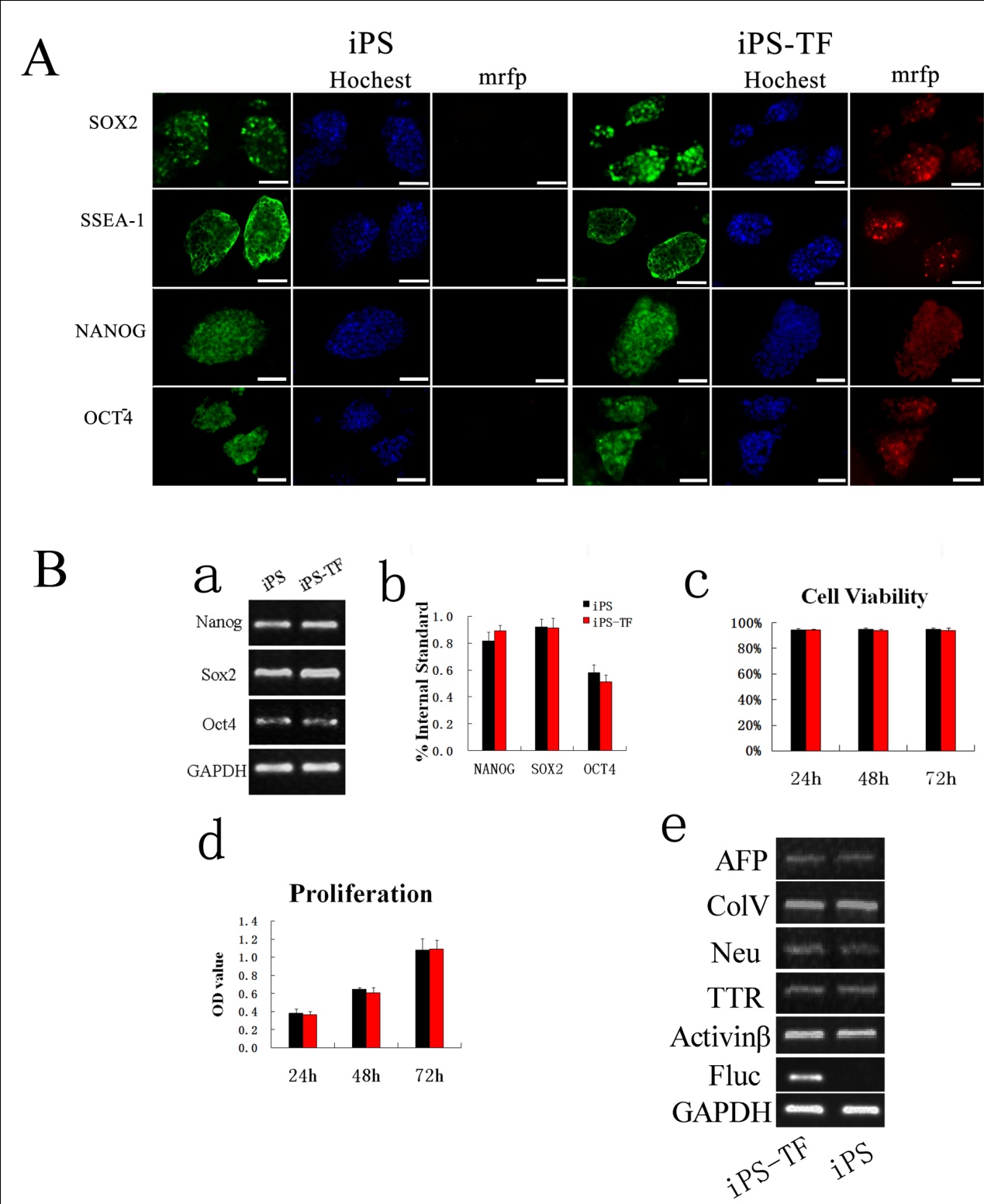
**

**FigS1. Characterization and Comparison between control iPS and iPS-TF cells.** Both iPS and iPS-TF colonies cells were positive for pluripotent ESC markers -- SOX2, SSEA-1, NANOG and OCT4. In iPS-TF colonies, colocalization of mrfp and ESC markers were observed under a fluorescent microscope, while in iPS colonies, no expression of mrfp was observed. The nucleus were stained with hochest (Bar=100μm) (A). The expression of ESC markers were also analyzed in gene level by RT-PCR, no significant difference were observed between the control iPS and iPS-TF cells(B-a, B-b); Cell viabilities and proliferation of iPS and iPS-TF cells were analyzed and compared within 72h culture. Quantification of viable cells at 24h, 48h and 72h time points showed no significant difference between iPS and iPS-TF cells(B-c); No significant difference in proliferation capacities was observed between iPS and iPS-TF cells either(B-d); After EB formation and differentiation, both iPS and iPS-TF cells could differentiate into tridermic lineages(B-e).


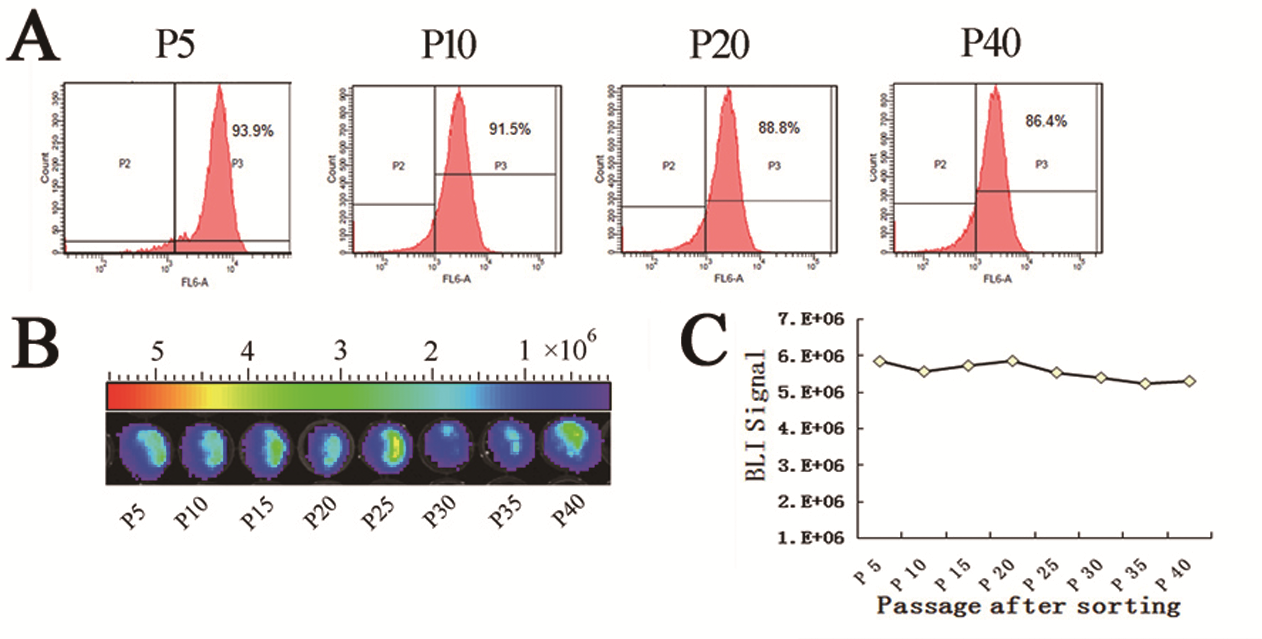


**FigS2. Stable expression of repoter gene in iPSC-TF cells.** (A), Stable expression of repoter gene during long-term passage of iPSCs; (B, C), Bioluminescence assay on the same number (10^5^) of iPSC-TF cells from different passages;


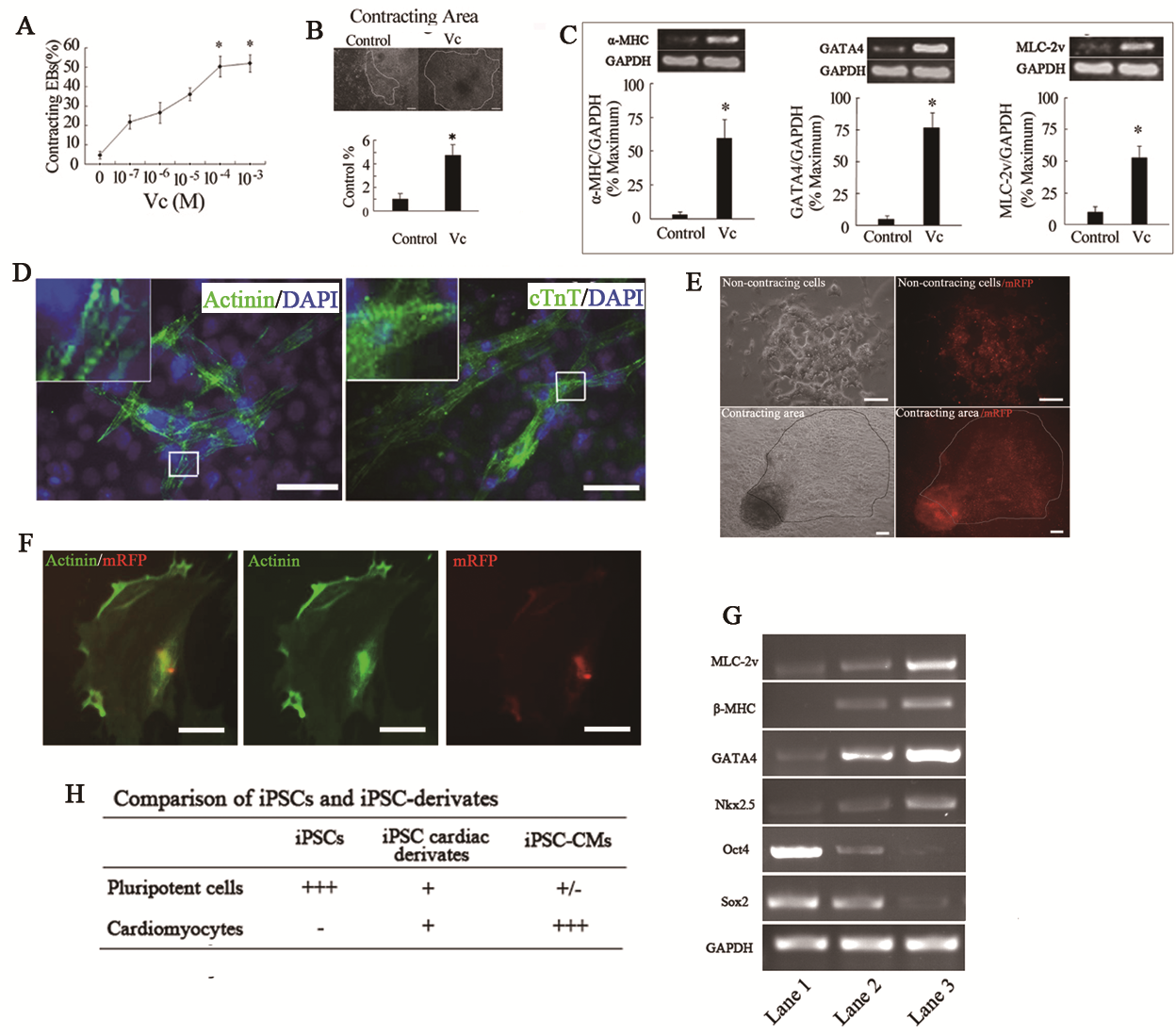


**FigS3. Differentiation and characterization of iPSC-CMs.**  (A), Comparison of the contracting areas (Bar=100μm). (C) The expression of cardiac genes in Vc-induced/spontaneous differentiation of iPSCs; (D) Contracting EBs were positively stained by cardiac-specfic antibodies. (Bar=50μm). (E) Expression of reporters in iPSC-noncontracting and contracting derivates; (F), Expression of reporters in iPSC-CMs(Bar=50μm); (G) The expression of pluripotent, cardiac genes in iPSCs, iPSC-cardiac derivates and iPSC-CMs. Lane 1: iPSCs; Lane 2: iPSC-cardiac derivates; Lane 3: iPSC-CMs; (H) Comparison of iPSCs, iPSC-cardiac derivates and iPSC-CMs. **p*<0.01.

**References**

1 Cao, F., Lin, S., Xie, X., Ray, P., Patel, M., Zhang, X., Drukker, M., Dylla, S.J., Connolly, A.J., Chen, X., Weissman, I.L., Gambhir, S.S., and Wu, J.C. (2006). In vivo visualization of embryonic stem cell survival, proliferation, and migration after cardiac delivery. Circulation *113*, 1005-1014.

2 Swijnenburg, R.J., Tanaka, M., Vogel, H., Baker, J., Kofidis, T., Gunawan, F., Lebl, D.R., Caffarelli, A.D., de Bruin, J.L., Fedoseyeva, E.V., and Robbins, R.C. (2005). Embryonic stem cell immunogenicity increases upon differentiation after transplantation into ischemic myocardium. Circulation *112*, I166-172.
